# Identification of QTL and candidate genes associated with biomass yield and Feed Quality in response to water deficit in alfalfa (*Medicago sativa* L.) using linkage mapping and RNA-Seq

**DOI:** 10.3389/fpls.2022.996672

**Published:** 2022-10-17

**Authors:** Xueqian Jiang, Andong Yu, Fan Zhang, Tianhui Yang, Chuan Wang, Ting Gao, Qingchuan Yang, Long-Xi Yu, Zhen Wang, Junmei Kang

**Affiliations:** ^1^ Institute of Animal Science, Chinese Academy of Agricultural Sciences, Beijing, China; ^2^ Institute of Animal Science, Ningxia Academy of Agricultural and Forestry Sciences, Ningxia, China; ^3^ Plant Germplasm Introduction and Testing Research, United States Department of Agriculture-Agricultural Research Service, Prosser, WA, United States

**Keywords:** alfalfa, biomass yield, Feed Quality, drought, linkage mapping, RNA-Seq

## Abstract

Biomass yield and Feed Quality are the most important traits in alfalfa (*Medicago sativa* L.), which directly affect its economic value. Drought stress is one of the main limiting factors affecting alfalfa production worldwide. However, the genetic and especially the molecular mechanisms for drought tolerance in alfalfa are poorly understood. In this study, linkage mapping was performed in an F1 population by combining 12 phenotypic data (biomass yield, plant height, and 10 Feed Quality-related traits). A total of 48 significant QTLs were identified on the high-density genetic linkage maps that were constructed in our previous study. Among them, nine main QTLs, which explained more than 10% phenotypic variance, were detected for biomass yield (one), plant height (one), CP (two), ASH (one), P (two), K(one), and Mg (one). A total of 31 candidate genes were identified in the nine main QTL intervals based on the RNA-seq analysis under the drought condition. Blast-P was further performed to screen candidate genes controlling drought tolerance, and 22 functional protein candidates were finally identified. The results of the present study will be useful for improving drought tolerance of alfalfa varieties by marker-assisted selection (MAS), and provide promising candidates for further gene cloning and mechanism study.

## Introduction

Abiotic stresses, such as drought, cold, and salinity, are misfortunes for agriculture, which seriously limit crop productivity. In particular, drought is an increasing worldwide threat (Gupta et al., 2020). Economic losses in crop production were almost $30 billion in the past decade due to drought stress (Gupta et al., 2020). By 2050, water demand for agriculture could be double and economic losses in crop production due to water scarcity could be higher owing to climate change (Gleick, 2000; Tang, 2019; Gupta et al., 2020).

Alfalfa, the “Queen of Forages”, has a capacity to produce high yields and high-quality forage. The dry matter yield of alfalfa ranges from 12 to 19 t ha^-1^ (Nešić et al., 2005). On the other hand, alfalfa is a rich in protein, with over 20% crude protein content in alfalfa hay (Long et al., 2022). Furthermore, alfalfa is well recognized for its high concentration of macroelements (such as N, P, and Ca), microelements (such as Fe, Zn, Cu, and Mn), and vitamins (such as A, E, and K), all of which are beneficial to animal health (Radović et al., 2009). All those characteristics confirm that alfalfa has a superior value in feeding animals. However, alfalfa production and yield stability are severely affected by drought (Ashrafi et al., 2018; Ines et al., 2022). It has been reported that severe drought causes severe economic losses (Nadeem et al., 2019). Slama et al. (2011) studied eight cultivars of *Medicago sativa* and found that the biomass production was reduced by 55–75% for plants subjected to a water deficit condition (Slama et al., 2011). As a result, developing alfalfa cultivars with improved water use efficiency (WUE) and drought tolerance is critical for sustainable alfalfa production in water-limited areas.

Traditional breeding programs to develop new alfalfa varieties are time-consuming and costly. Incorporating marker-assisted selection (MAS) into breeding programs holds one of the promises to meet the demand for alfalfa production. Although, MAS has been widely used in several important crops, such as maize (Samayoa et al., 2019), wheat (Kumar et al., 2018a), and soybean (Kim et al., 2020). It is rarely employed for the commercial development of improved alfalfa varieties. Identifying and developing genetic loci robustly associated with alfalfa drought tolerance is the first step to developing drought resistant varieties by MAS. Quantitative trait loci (QTL) mapping and genome-wide association studies (GWAS) have been used to identify QTLs that influence complex quantitative traits, such as drought resistance. To date, some QTLs/SNPs for drought resistance have been detected by linkage or association mapping. For instance, Ray et al. (2015) identified 10 and 15 QTL associated with increased or reduced forage yield during drought stress in two backcross (BC1) mapping populations. Santantonio et al. (2019) performed linkage mapping of forage yield, WUE, carbon and nitrogen metabolism in the same populations under drought conditions (Santantonio et al., 2019). A diversity panel of 198 alfalfa accessions was used to identify SNPs associated with drought tolerance using association mapping (Zhang et al., 2015; Yu, 2017; Lin et al., 2020). In a greenhouse, Zhang et al. (2015) identified 19 and 15 loci associated with drought resistance index and relative leaf water content, respectively. In the field, SNPs associated with biomass yield and 26 Feed Quality-related traits under water deficit have been identified in the same panel (Yu, 2017; Lin et al., 2020).

Functional genes that contribute to drought tolerance were identified using homology-based cloning, including *MsMYB2L* (Song et al., 2019), *MsZIP* (Li et al., 2013), *MsHSP17.7* (Li et al., 2016), *MsZEP* (Zhang et al., 2016), *MsHSP70* (Li et al., 2017b), *MsCML46* (Du et al., 2021), *MsVDAC* (Yang et al., 2021b), *MsWRKY11* (Wen et al., 2021). However, little is known about genetic factors that contribute to drought tolerance in alfalfa. In the present study, we evaluated biomass yield, plant height, and 10 Feed Quality traits in an F1 mapping population under water deficit. Linkage mapping and RNA-sequencing (RNA-seq) analyses were performed to identify the QTLs associated with drought tolerance, and candidate genes by screening the differentially expressed genes (DEGs) within the QTL intervals. The detected QTLs are valuable resources for alfalfa genetic improvement by MAS, and further investigation of candidate genes can provide insights into genetic factors of alfalfa resistance to drought and other abiotic stresses.

## Materials and methods

### Mapping population development, genotyping, and genetic linkage maps

The F1 population consisting of 150 progenies was used for evaluating and identifying loci associated with drought tolerance. Mapping population development, genotyping, and construction of genetic linkage maps were provided in our previous study (Jiang et al., 2022). Briefly, the mapping population was developed by crossing two tetraploid alfalfa plants, Cangzhou (CF000735, paternal parent, P1) and Zhongmu NO.1 (CF0032020, maternal parent, P2). The two parents vary in drought tolerance capacity with P1 better than P2. In greenhouse, both parents were imposed to water stress at 40% field capacity for 4 weeks, while control pots maintained 100% field capacity throughout the period of regrowth cycle. The biomass yield of plants under drought stress decreased significantly by 27.4% and 47.3%, as compared to control plants for P1 and P2, respectively. According to the method of Li et al. (2014), alleles segregating with a ratio of less than 2:1 were considered as single-dose allele (SDA) markers. A total of 7,252 and 7,404 high-quality SDA markers were obtained for P1 and P2 parents, respectively; which were then used to construct high-density linkage maps by JoinMap 4.0 software (Van Ooijen, 2006).

### Field experiments and phenotyping

In 2016, cloned plants of the F1 population and two parents were transplanted to the field of the Chinese Academy of Agricultural Sciences Research Station at Langfang, Hebei Province (39.59°N, 116.59°E). The field experiment used a randomized complete block design with three replications, where one cloned plant of 150 progenies and two parents were planted in each replication. At the field site, the rainfall during the three alfalfa first growth cycles of 2018, 2019, and 2020 was approximately 24.7, 35.6, and 77.8mm, which was far less than the water requirement for normal growth of alfalfa (Cole et al., 1970; Dobrenz et al., 1971). Drought treatment was applied to the plants by withholding water before the first cut. Thus, three forage regrowth cycles (the first cut of 2018, 2019 and 2020) experienced significant water stress. In the early flowering stage, we measured biomass yield (BY), plant height (PH), and ten quality-related traits, including: the content of crude protein (CP), neutral detergent fiber (NDF), acid detergent fiber (ADF), lignin, dry matter (DM), ASH, K, Ca, Mg, and P. Plant height was the length of the longest stem, and biomass was the plant’s fresh weight when the stubble height is 4 ~ 5 cm. The BY and PH of individual plants were measured when the first flower appeared. The Feed Quality was measured using near-infrared reflectance spectroscopy (Foss NIRS 1650), and the details were described by Yang et al. (2021a). Statistical analysis of 12 traits was estimated by the R package psych. The best linear unbiased estimation (BLUE) and broad-sense heritability (H^2^) of the 12 traits were estimated by the ANOV function in the IciMapping software (Meng et al., 2015).

### Linkage mapping

Combining BLUE values of each trait and the genetic linkage maps, QTL in response to water deficit were identified in the mapping population using the Inclusive Composite Interval Mapping with an additive effect (ICIM-ADD) in QTL IciMapping software (Meng et al., 2015). The QTL with a LOD value ≥ 3.0 was selected as significant QTL. QTLs were named as: q + phenotype + linkage group no., or qFT + linkage group no. + an ordered number to designate QTL in a single linkage group. For example, *qBY6.4-1* indicates the first QTL associated with biomass yield under a water deficit condition in the Chr6.4 linkage group.

### RNA-seq analysis

In brief, RNA-seq sequences were filtered using fastp (Chen et al., 2018), and mapped to the XingJiangDaYe reference genome (Chen et al., 2020b) using hisat2 (Kim et al., 2019). Samtools was used to generate and sort BAM files (Li et al., 2009). FeatureCounts v2.0.1 was used to generate read counts for each sample (Liao et al., 2014). The FPKM (Fragments Per Kilobase Million) was utilized to normalize and estimate gene expression values. The |log2 (FoldChange)| ≥ 2 and *P* ≤ 0.01 were used as thresholds to assess the significance of gene expression difference. TBtools was used to do a Gene Ontology (GO) enrichment analysis of the differentially expressed genes. (Chen et al., 2020a). Three RNA-sequencing datasets were used for the discovery of differentially expressed candidate genes associated with drought tolerance within the QTL region. The datasets were submitted to NCBI and signed for project numbers as follow: The first dataset, PRJNA525327 (https://www.ncbi.nlm.nih.gov/bioproject/PRJNA525327/), was an RNA-seq transcriptome profiling of two alfalfa genotypes (drought-sensitive and drought-resistant) root tissue under PEG-induced drought stress. The second one, PRJNA765383 (https://www.ncbi.nlm.nih.gov/bioproject/PRJNA765383/), was an RNA-seq of two genotypes (drought-sensitive and drought-resistant) of alfalfa leaf under drought conditions. The third RNA-seq project (PRJNA450305, https://www.ncbi.nlm.nih.gov/bioproject/PRJNA450305/) was alfalfa seedlings (Zhongmu No.1) treated with 400 mM mannitol under different treatment time points (0, 12, and 24 h).

### Prediction of candidate genes

The flanking markers of main QTLs, which explained more than 10% of the phenotypic variance, were used to obtain the physical location of QTLs on the XingJiangDaYe reference genome. The genes located on the flanking markers and within the physical intervals were extracted for further analysis. The first two RAN-seq datasets resulted in four comparative groups (G1~G4). The extracted genes, which were differentially expressed in two or more comparative groups, were used to further identify if they were differentially expressed in different treatment time points in the third RNA-seq project. If a candidate is identified by combining the information on linkage mapping and RNA-seq analysis, then, it will be annotated based on BLSAT search in Ensembl (https://ensembl.gramene.org/).

Quantitative real-time PCR (qRT-PCR) was performed to confirm whether the expression of candidate genes was induced by drought stress. Alfalfa seeds (Zhongmu No.1) were germinated in the MS medium. After seven days germination, the seedlings with similar growth were transferred to a hydroponic pot filled with 1/2 MS nutrient solution (pH = 5.8). Ten days after transfer, PEG-6000 (20%) was used to simulate drought treatment. Total RNA for each sample was isolated from three palnts under a time course of drought treatments (0, 1, 3, 6, 12, and 24 h). Using Ms-actin gene as the reference gene, qRT-PCR was implemented in triplicate for each treatment with the 7500 Real-Time PCR System (Applied Biosystem, CA, USA). The relative gene expression level was calculated by the 2-△△Ct method.

## Results

### Phenotypic data analysis

The BLUE values of the 12 traits including yield, plant height and 10 quality-related traits were used for statistical analysis. Two parents appeared to have substantial variations in these measured traits except for ADF and DM (**Table S1**). For example, the BY of the P2 parent was significantly higher than that of the P1 parent. A frequency distribution histogram based on the F1 population revealed a nearly normal distribution for 12 traits, indicating that the traits measured were quantitative traits (**Figure S1**). As shown in **Figure S2**, a positive correlation was observed between BY and PH in the F1 population with a correlation coefficient of 0.69 (**Figure S2**), indicating that the genotypes with higher PH tended to have higher yields. For quality related traits, CP was positively correlated with lignin (0.34, *P* < 0.001), Ca (0.48, *P* < 0.001), P (0.74, *P* < 0.001), and K (0.65, *P* < 0.001), while negatively correlated with Mg (-0.27, *P* < 0.01), ADF (−0.44, *P* < 0.001), and NDF (-0.37, *P* < 0.001). For lignin, it was positively correlated with ADF (0.55, *P* < 0.001) and NDF (0.57, *P* < 0.001) (**Figure S2**). The correlations we observed were consistent with those in the previous reports (Yang et al., 2021a).

The H^2^ of the 12 traits in the F1 population have a high variation ranging from 0.28 to 0.61 (**Table S1**). BY and PH had higher heritability of 0.50 and 0.61, respectively, suggesting that high proportion of the variability of these traits were contributed by genetic factor. Among the 10 Feed Quality-related traits, DM and ADF had the lowest heritability, at 0.28 and 0.32, respectively, indicating that a high proportion of the variability in these traits came from environmental factors, with a smaller contribution from genetic differences.

### QTL analysis

Under water deficit, BY, PH, and ten quality-related traits of the alfalfa population were evaluated during three consecutive years from 2018 to 2020. Using a threshold of LOD value higher than 3, a total of 48 QTLs were identified with phenotypic variance explanations ranging from 2.93% to 16.03% (**Table 1**). For two yield related traits, BY and PH, we identified 11 QTLs in two parents with six for BY and five for PH. We detected 37 QTLs related to ten quality traits with 8, 1, 3, 7, 1, 9, 3, and 5 for content of CP, lignin, NDF, ASH, Ca, P, K, and Mg, respectively, while, no QTL was detected for ADF and DM (**Table 1**).

**Table 1:** QTLs detected for 12 traits using ICIM-ADD in the F1 population.

Parent	QTL	LG	Position/cM	LeftMarker	RightMarker	LOD	PVE (%)
P1	*qBY1.1*	chr1.1	8.5~9.5	chr1.1:1188379	chr1.1:25288609	6.95	6.62
P1	*qBY1.4*	chr1.4	45.5~46.5	chr1.4:32618614	chr1.4:38011048	4.70	5.78
P1	*qBY2.1*	chr2.1	91.5~92.5	chr2.1:40634643	chr2.1:51966907	3.68	2.93
P1	*qBY3.3*	chr3.3	34.5~35.5	chr3.3:39725093	chr3.3:51651121	6.60	6.22
P1	*qBY6.4-1*	chr6.4	67.5~68.5	chr6.4:19754452	chr6.4:38317952	6.01	8.85
P2	***qBY6.4-2***	chr6.4	9.5~10.5	chr6.4:11679540	chr6.4:13292372	3.16	10.13
P1	*qPH6.1*	chr6.1	68.5~69.5	chr6.1:62112875	chr6.1:59533246	3.83	9.03
P1	***qPH7.3***	chr7.3	83.5~84.5	chr7.3:63690328	chr7.3:63690347	7.30	16.03
P2	*qPH3.1*	chr3.1	56.5~57.5	chr3.1:35511599	chr3.1:24556189	3.63	8.16
P2	*qPH5.4*	chr5.4	46.5~47.5	chr5.4:15100953	chr5.4:24519386	3.73	7.38
P2	*qPH7.1*	chr7.1	96.5~97.5	chr7.1:70529293	chr7.1:62826189	4.26	8.83
P1	*qCP1.4-1*	chr1.4	4.5~5.5	chr1.4:4781072	chr1.4:18320659	6.73	6.61
P1	*qCP1.4-2*	chr1.4	56.5~57.5	chr1.4:40757995	chr1.4:68168405	4.48	7.30
P1	*qCP2.3*	chr2.3	28.5~29.5	chr2.3:25484901	chr2.3:25484816	4.33	4.37
P1	***qCP3.3***	chr3.3	77.5~78.5	chr3.3:82448563	chr3.3:85819420	8.73	11.13
P1	*qCP4.2*	chr4.2	81.5~82.5	chr4.2:83498816	chr4.2:86046636	6.25	6.47
P1	*qCP5.3*	chr5.3	66.5~67.5	chr5.3:47283990	chr5.3:56958651	6.29	7.40
P1	*qCP6.3*	chr6.3	45.5~46.5	chr6.3:24707110	chr6.3:13214112	3.61	5.88
P2	***qCP3.4***	chr3.4	78.5~79.5	chr3.4:51603347	chr3.4:51603328	4.53	10.77
P1	*qlignin6.4*	chr6.4	38.5~39.5	chr6.4:13303817	chr6.4:15215403	4.63	4.08
P2	*qNDF5.1*	chr5.1	46.5~47.5	chr5.1:32987072	chr5.1:48353467	4.62	6.04
P2	*qNDF6.4*	chr6.4	105.5~111.5	chr6.4:61366096	chr6.4:60044123	3.43	5.96
P2	*qNDF8.4*	chr8.4	53.5~54.5	chr8.4:54287851	chr8.4:58806497	5.26	9.14
P1	***qASH5.4***	chr5.4	61.5~62.5	chr5.4:42741251	chr5.4:37142807	3.79	10.70
P1	*qASH8.1*	chr8.1	65.5~66.5	chr8.1:57462848	chr8.1:64832251	3.43	6.12
P2	*qASH1.2*	chr1.2	33.5~34.5	chr1.2:29902008	chr1.2:30572221	3.97	4.00
P2	*qASH1.4*	chr1.4	117.5~118.5	chr1.4:66910982	chr1.4:82180569	8.63	9.27
P2	*qASH4.4*	chr4.4	89.5~90.5	chr4.4:66438303	chr4.4:60823700	7.07	7.33
P2	*qASH5.3*	chr5.3	46.5~48.5	chr5.3:9887846	chr5.3:12938236	3.39	4.24
P2	*qASH8.2*	chr8.2	94.5~95.5	chr8.2:76204355	chr8.2:83554323	6.27	5.86
P1	*qCa4.3*	chr4.3	4.5~7.5	chr4.3:405639	chr4.3:3983154	3.53	9.29
P1	*qP2.4*	chr2.4	59.5~60.5	chr2.4:35238369	chr2.4:33943447	3.87	7.38
P1	*qP4.1*	chr4.1	22.5~23.5	chr4.1:35458256	chr4.1:28488544	4.55	5.71
P1	***qP4.2***	chr4.2	81.5~82.5	chr4.2:83498816	chr4.2:86046636	10.45	14.01
P1	*qP5.2*	chr5.2	56.5~57.5	chr5.2:70405584	chr5.2:79796969	4.42	6.07
P1	*qP7.4*	chr7.4	64.5~65.5	chr7.4:56305064	chr7.4:74538855	3.29	5.38
P2	*qP2.2*	chr2.2	91.5~92.5	chr2.2:46344535	chr2.2:68020962	3.31	4.80
P2	*qP3.4*	chr3.4	78.5~79.5	chr3.4:51603347	chr3.4:51603328	5.66	9.32
P2	*qP7.1-1*	chr7.1	5.5~7.5	chr7.1:15149480	chr7.1:15158721	3.95	7.53
P2	***qP7.1-2***	chr7.1	34.5~35.5	chr7.1:18573847	chr7.1:21230294	5.55	10.72
P2	*qK1.1*	chr1.1	28.5~29.5	chr1.1:29260583	chr1.1:26628171	5.32	8.08
P2	*qK5.3*	chr5.3	148.5~152.5	chr5.3:79503684	chr5.3:79503581	4.49	5.83
P2	***qK8.2***	chr8.2	18.5~19.5	chr8.2:28815850	chr8.2:26672846	8.01	14.00
P2	*qMg1.1*	chr1.1	45.5~46.5	chr1.1:53165176	chr1.1:46575248	3.11	3.52
P2	*qMg1.4*	chr1.4	14.5~17.5	chr1.4:4062805	chr1.4:4058912	4.17	2.98
P2	*qMg3.4*	chr3.4	85.5~86.5	chr3.4:68267075	chr3.4:73873783	7.09	6.98
P2	***qMg4.3***	chr4.3	118.5~119.5	chr4.3:85323781	chr4.3:80402482	12.24	13.43
P2	*qMg7.3*	chr7.3	13.5~14.5	chr7.3:24844583	chr7.3:4955198	5.68	4.89

A total of 48 QTLs were mapped by best linear unbiased estimation (BLUE) values on P1 and P2 linkage maps. Main QTLs (PVE > 10%) were bold. LG, linkage group; Position/cM, 1-LOD

support interval; Leftmarker, the marker on the left of the LOD peak; Rightmarker, the marker on the right of the LOD peak; LOD, the logarithm of the odds; PVE, the percentage of the

phenotypic variation explained by QTL.

**Figure 1:**
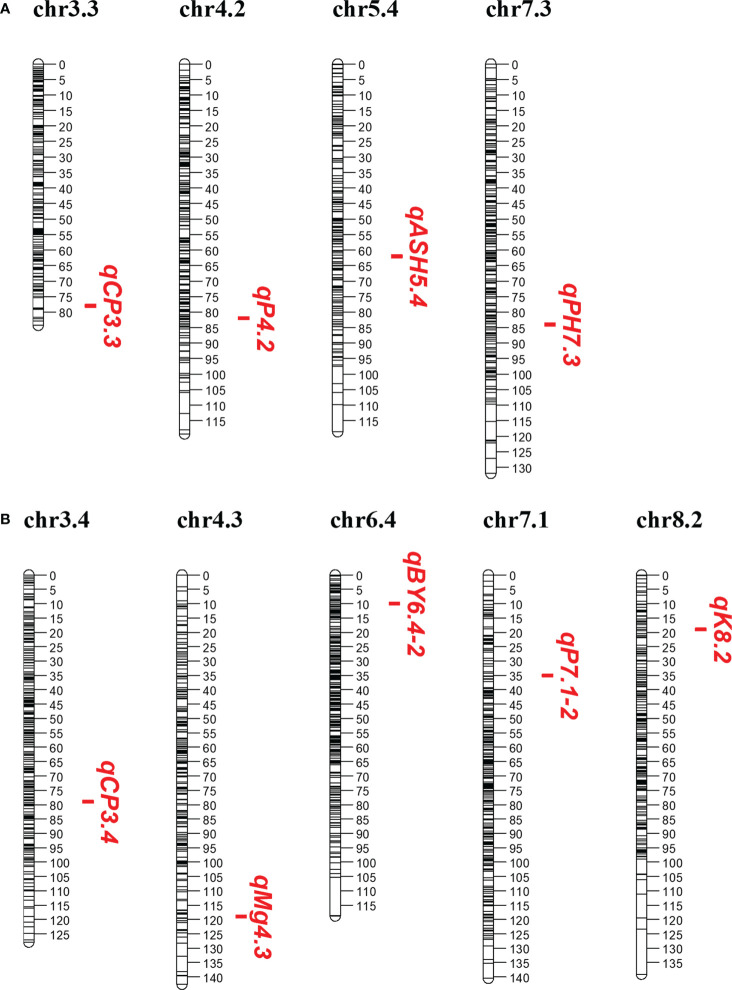
Main QTLs (PVE > 10%) detected in this study. **(A, B)** Main QTLs in the P1 and P2 parent, respectively.

Among these significant QTLs, nine showed more than 10% of the phenotypic variation explained (PVE) individually, with four in P1 and five in P2 (**Table 1**; **Figure 1**). PVE by four main QTLs, qPH7.3, qCP3.3, qASH5.4, and qP4.2, ranged from 10.70 to 16.03% in the P1 parent, with the highest PVE on qPH7.3 and the lowest PVE on qASH5.4 (**Table 1**; **Figure 1A**). In the P2 parent, five main QTLs of *qBY6.4-2*, *qCP3.4*, *qP7.1-2*, *qK8.2*, and *qMg4.3* explained 10.13, 10.77, 10.72, 14.00, and 13.43% of the phenotypic variation, respectively (**Table 1**; **Figure 1B**). For BY, one main QTL, *qBY6.4-2*, was located on Chr6.4, while one PH-related main QTL, *qPH7.3*, was located on Chr7.3 with a LOD score of 7.03. For ten Feed Quality traits, seven main QTL were identified, including: two for CP, one for ash, two for P, one for K, and one for Mg. In contrast, no main QTL was detected for lignin, ADF, NDF, DM, and Ca (**Table 1**).

Among the nine main QTLs, three QTLs from P1 and three from P2 were also independently identified in single environment (**Table S2**). They were considered as consistent QTLs. Specifically, *qCP3.3*, *qCP3.4*, and *qK8.2* were also identified in 2019, 2020, and 2020, respectively. *qBY6.4-2* and *qPH7.3* were detected in two years. The last one, *qP4.2*, was identified in three grown environments (2018, 2019, and 2020) at a position of 81.5 ~ 82.5 cM.

### Analysis of DEGs

**Figure 2:**
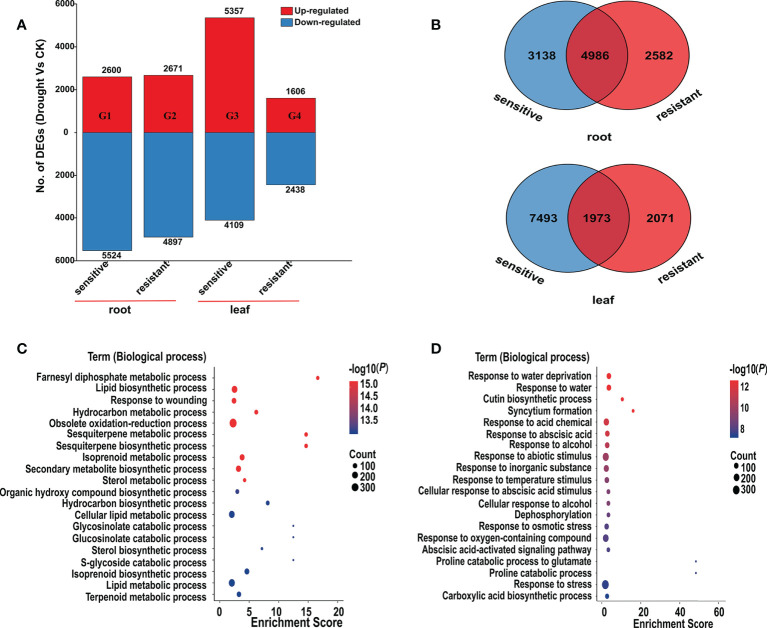
Differentially expressed genes (DEGs) in root and leaf. **(A)**, Number of upregulated genes (red) and downregulated genes (blue) in four comparative groups (G1 ~ G4). You can see the specific information of these four comparative groups in the **Table S3**. **(B)**, Venn diagram of DEGs among root and leaf. **(C, D)**, Bubble chart of GO enrichment analysis of the DEGs in root **(C)** and leaf **(D)**. The ordinate represents different GO terms (biological progress) and the abscissa represents enrichment Score. Circle size represents the gene number while circle color represents the value of −log10 (*P*). The *P* value was corrected by Benjamini & Hochberg (BH) method.

In order to elucidate the drought-induced transcripts in alfalfa, we performed transcriptome analysis in leaf and root. Four groups (G1 ~ G4) were constructed by comparing the same genotype of root and leaf tissues under different conditions (control and drought) (**Figure 2A**; **Tables S3** , **S4**). In root, the number of DEGs (drought vs. control) was approximately equal in the two comparative groups. There were 2,600 genes that were up-regulated in G1, while 2,671 genes were up-regulated in G2. Meanwhile, 5,524 and 4,897 down-regulated genes were identified in G1 and G2, respectively (**Figure 2A**). In contrast, compared to the 1,606 up- and 2,438 down-regulated DEGs identified in the G4, 5,357 up- and 4,109 down-regulated DEGs were identified in G3, indicating that the drought responsive transcriptomic program in leaf had bigger difference than those in root in different alfalfa genotypes (**Figure 2A**). In drought-resistant genotypes, drought condition upregulated 1,606 and 2,671 genes, and downregulated 2,438 and 4,897 genes in the leaf (G4) and root (G2) respectively. Not surprisingly, drought conditions resulted in more common DEGs in root, with 4,986 in root and 1,973 in leaf (**Figure 2B**).

**Figure 3:**
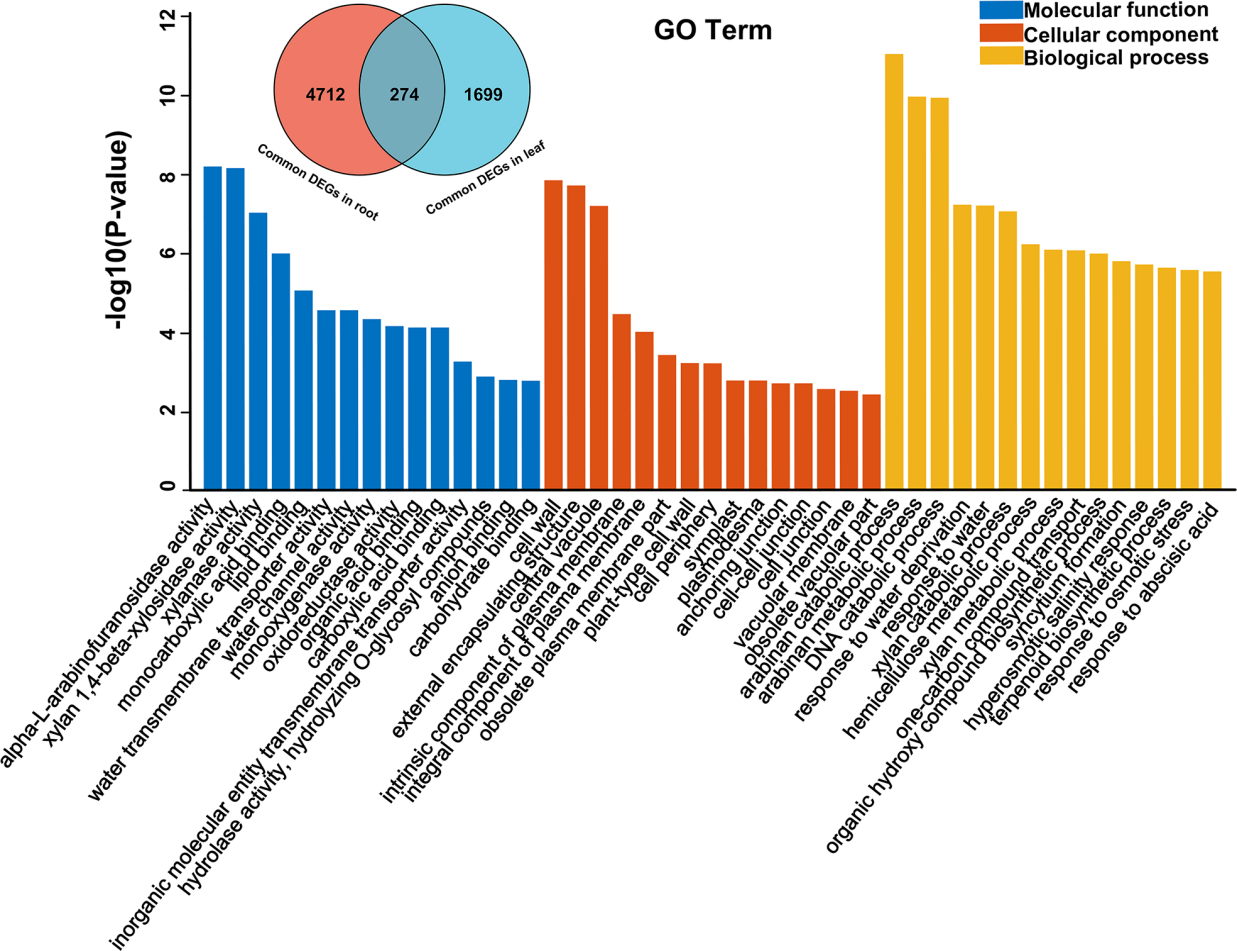
GO enrichment bar chart of 274 common DEGs among root and leaf.

Through GO enrichment analysis, the common DEGs in alfalfa root and leaf were then classified into the biological process (BP), cellular component (CC), and molecular function (MF) (**Figures 2C, D**; **Tables S5** , **S6**). The common DEGs in root were enriched in the BP terms “response to acid chemical”, “monocarboxylic acid biosynthetic process”, “monocarboxylic acid biosynthetic process”, “oxoacid metabolic process”, “organic acid metabolic process”, “organic hydroxy compound biosynthetic process” and so on (**Figure 2C**; **Table S5**). These GO terms were also enriched in leaf (**Table S6**), indicating that these common genes were possibly involved in responding to drought stress. Subsequent GO enrichment analysis of 274 common DEGs in all four comparative groups resulted that these DEGs had a role in “response to water deprivation”, “response to osmotic stress” and “response to abscisic acid” and other responses (**Figure 3**, **Table S7**). Based on their functions, these genes likely play crucial roles in drought tolerance.

### Integration of DEGs with QTLs

Linkage mapping revealed nine major QTL (PVE > 10%), which were selected as genetic regions to identify candidate genes responsive to drought tolerance. We first located their physical positions on the XingJiangDaYe reference genome. For instance, two physical intervals of *qPH7.3* and *qCP3.4* were less than 1Mb. We therefore screened candidate genes within 1cM upstream and downstream of these two QTL flanking markers. In this way, a total of 1,455 genes were extracted from the nine main QTL intervals (**Table 2**). Among them, three genes (*MS.gene013861*, *MS.gene065379*, and *MS.gene034021*) were located on flanking markers. *chr4.3:85323781*, the left flanking markers of *qMg4.3*, was lined with *MS.gene034021*, which was differentially expressed in G1 and G2 (**Table S8**).

**Table 2:** Number of genes, DEGs within nine main QTL (PVE > 10%) regions.

QTL	Genes		DEGs						
		Root			Leaf			^c^Common
		G1	G2	^a^Common	G3	G4	^b^Common	
*qCP3.3*	280	24	21	15	15	6	3	0
*qCP3.4*	97	3	4	3	5	2	1	0
*qP4.2*	138	8	14	8	8	2	2	2
*qMg4.3*	273	15	16	9	19	12	8	2
*qASH5.4*	149	10	6	5	11	5	4	1
*qBY6.4-2*	60	2	4	1	4	1	0	0
*qP7.1-2*	223	11	11	6	19	7	3	0
*qPH7.3*	75	2	3	2	3	0	0	0
*qK8.2*	160	7	4	3	12	2	1	0
Total	1455	82	83	52	96	37	22	5

^a^Common DEGs among G1 and G2.

^b^Common DEGs among G3 and G4.

^c^Common DEGs among four comparative groups (G1 ~ G4).

**Figure 4:**
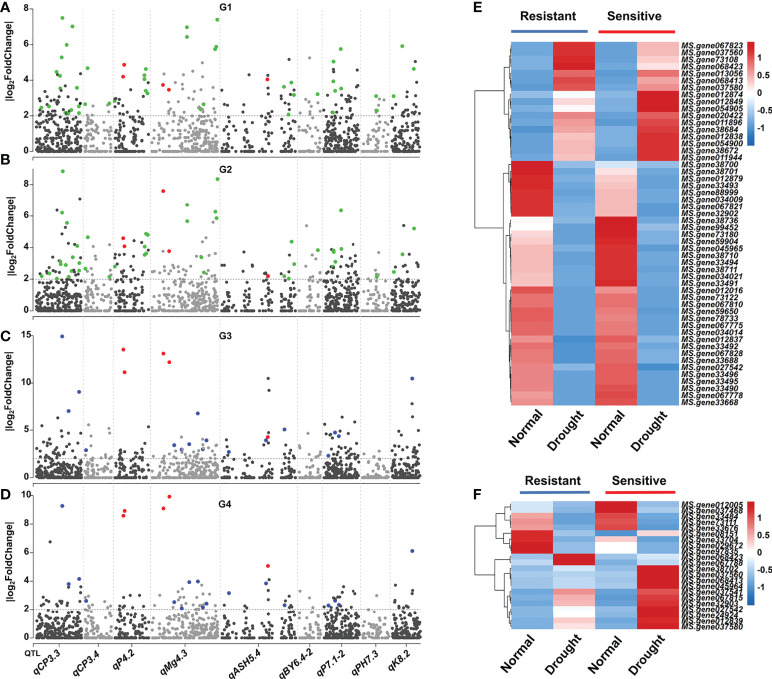
Combined transcriptome analysis and linkage mapping revealing the potential causal genes for drought response in alfalfa. **(A–D)** Distribution of DEGs within nine main QTL regions in four comparative groups, respectively. Green, blue, and red colors represent common DEGs among root, leaf, and four comparative groups, respectively. **(E)**, Heatmap of 52 common DEGs among root. **(F)** Heatmap of 22 common DEGs among leaf.

To detect the candidate genes within the nine intervals, we combined the linkage mapping and RNA-seq analysis results. For G1, a total of 82 DEGs located in the nine main QTL regions, and the number of DEGs within each QTL ranged from 2 (*qBY6.4-2*/*qPH7.3*) to 24 (*qCP3.3*) (**Table 2**; **Figure 4A**). Also, 83, 80, and 37 DEGs located in the nine main QTL regions for G2, G3, and G4, respectively (**Table 2**; **Figures 4A–D**). Additionally, there were 52 common DEGs in roots (G1 and G2) and 22 common DEGs located in the nine QTL regions in leaves (G3 and G4) (**Table 2**; **Figures 4E, F**). Interestingly, the abovementioned DEGs in leaves had different expression patterns. For example, *MS.gene08151* was down-regulated in drought condition in a drought-resistant genotype, while it was up-regulated in a drought-sensitive genotype (**Figure 4F**). However, the same phenomenon was not observed in root.

To obtain more evidence to determine the reliable candidate genes, we further analysis the expression pattern of these genes in drought conditions at different time points (0h, 12h, and 24h). Among them, 31 candidates within QTL regions were differentially expressed at different time points (**Figure S3**). Notably, five candidate genes were differentially expressed across four comparative groups and at different treatment time, of which two (*MS.gene068413* and *MS.gene068423*) were differentially expressed in *qP4.2*, two (*MS.gene037560* and *MS.gene037580*) in *qMg4.3*, and one (*MS.gene027542*) in *qASH5.4* (**Table 2**; **Figures 4A–D**).

**Figure 5:**
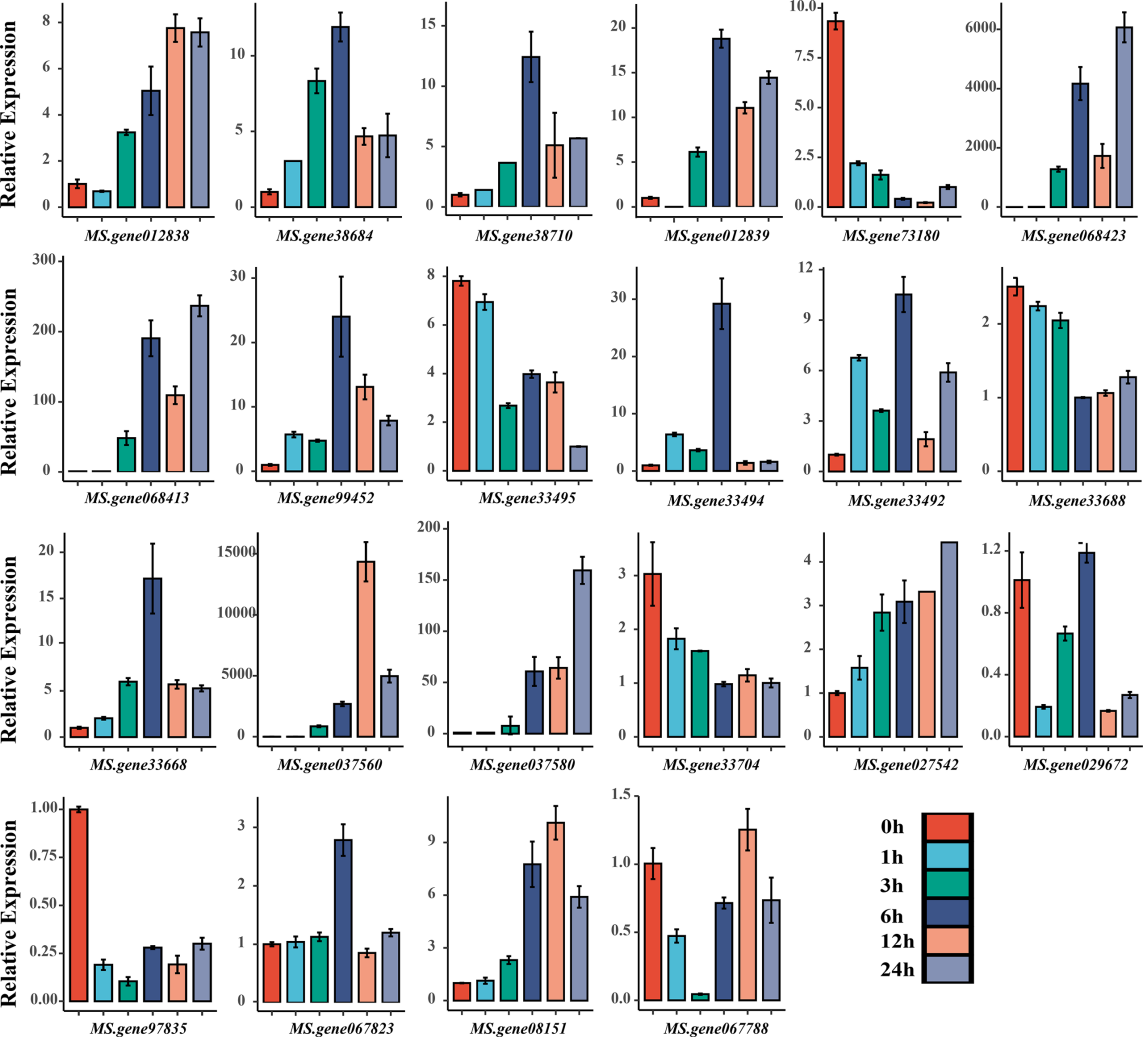
qRT-PCR analysis of the 22 candidate genes under drought stress.

Furthermore, functional annotation of the 31 identified genes revealed that 22 of them may be involved in drought stress regulation (**Table 3**). We investigated the expression level of all 22 candidate genes by qRT-PCR analysis, and found that all candidate genes were significantly induced under drought stress compared with untreated control (**Figure 5**). The results revealed that 13 candidate genes were consistent with the transcriptome results under drought stress with eight up-regulated genes (*MS.gene012838*, *MS.gene38684*, *MS.gene012839*, *MS.gene068423*, *MS.gene068413*, *MS.gene037560*, *MS.gene037580*, *MS.gene067823*) and five down-regulated genes (*MS.gene73180*, *MS.gene33495*, *MS.gene33688*, *MS.gene33704*, *MS.gene97835*) (**Table S8**; **Figure 5**). The expression patterns of the rest nine genes were different between the RNA-seq and qPT-PCR. For example, the expression level of *MS.gene99452* was down-regulated under drought stress in RNA-seq (**Table S8**). However, it was up-regulated in qRT-PCR with the highest expression level at 6 h after drought treatment (**Figure 5**). Differences in expression patterns may be due to differences in drought treatment conditions and the inherent genetic variation between the materials used for different experiments. These candidate genes identified in our study should be considered as putative candidates, further investigation should be performed.

**Table 3:** Potential candidate genes identified in this study.

QTL	GeneID	Position				BLAST-P	E-value	%ID
		Chr.	Start_Pos	End_Pos	Stand	protein_coding description	
*qCP3.3*	MS.gene012838	chr3.3	85656764	85658555	+	homeobox associated leucine zipper protein	4.6E-50	91.0
*qCP3.3*	MS.gene38684	chr3.3	84651967	84654396	+	Serine/Threonine kinase, plant-type protein	0	96.2
*qCP3.3*	*MS.gene38710*	chr3.3	84266981	84269413	+	ABA response element-binding factor	3.1E-114	99.5
*qCP3.3*	*MS.gene012839*	chr3.3	85634143	85642942	–	branched-chain amino acid aminotransferase	3.3E-126	97.9
*qCP3.4*	*MS.gene73180*	chr3.4	52454644	52454955	+	Lipid transfer protein	1.3E-51	94.9
*qP4.2*	*MS.gene068423*	chr4.2	84224298	84225247	–	phosphatidylethanolamine-binding protein	3.5E-111	95.3
*qP4.2*	*MS.gene068413*	chr4.2	84132136	84133091	–	phosphatidylethanolamine-binding protein	8.6E-113	96.4
*qP4.2*	*MS.gene99452*	chr4.2	83500593	83501671	+	PAR1 protein	3E-126	95.4
*qP4.2*	*MS.gene33495*	chr4.2	85748885	85750285	+	glycoside hydrolase family 1 protein	8.1E-119	95.5
*qP4.2*	*MS.gene33494*	chr4.2	85849862	85850722	+	glycoside hydrolase family 1 protein	2.4E-31	87.5
*qP4.2*	*MS.gene33492*	chr4.2	85977904	85980624	+	glycoside hydrolase family 1 protein	9.6E-144	91.3
*qMg4.3*	*MS.gene33688*	chr4.3	84072885	84075965	–	LRR receptor-like kinase family protein	0	95.5
*qMg4.3*	*MS.gene33668*	chr4.3	84295620	84297870	–	WRKY transcription factor	1.2E-154	97.8
*qMg4.3*	*MS.gene037560*	chr4.3	81683850	81684764	–	phosphatidylethanolamine-binding protein	5.2E-112	95.9
*qMg4.3*	*MS.gene037580*	chr4.3	81240143	81241108	–	UPF0098 protein CPn_0877	2E-118	95.8
*qMg4.3*	*MS.gene33704*	chr4.3	83826342	83826998	+	abscisic acid receptor	1.2E-123	95.3
*qASH5.4*	*MS.gene027542*	chr5.4	40643863	40645210	+	DUF1262 family protein	0	77.3
*qASH5.4*	*MS.gene029672*	chr5.4	37713550	37715701	–	myo-inositol oxygenase	0	97.8
*qASH5.4*	*MS.gene97835*	chr5.4	40503630	40505071	–	DUF538 family protein	1.2E-21	97.6
*qP7.1-2*	*MS.gene067823*	chr7.1	19308600	19310009	–	spermidine hydroxycinnamoyl transferase	0	93.2
*qP7.1-2*	*MS.gene08151*	chr7.1	18898187	18900692	–	plasma membrane H+-ATPase	4E-39	78.8
*qP7.1-2*	*MS.gene067788*	chr7.1	19687484	19688272	+	DUF4283 domain protein	1.4E-44	63.5

## Discussion

Analyzing the correlation coefficient in alfalfa forage yield and quality during drought stress can provide useful insight into phenotypic relationships between these traits in water-limited environments. To this end, we collected phenotypic data of two yield-related and 10 quality-related traits in the F1 mapping population for three years through drought-stressed growth cycle. P content was positively correlated with BY (0.34) and CP content (0.74), suggesting that applying P fertilizer may be beneficial to yield and CP content under drought conditions. For CP, it was negatively correlated (-0.37) with NDF, while the two traits had no significant correlation in a previous study (Yang et al., 2021a). Our results provided useful information to explore the relationship between alfalfa yield and quality during drought stress.

Achieving a high yield and high nutritional value is a main goal in alfalfa breeding. Forage yield and quality are easily influenced by the environment, such as drought stress. Previous studies suggested that biomass yield and Feed Quality under drought conditions may involve different mechanisms compared to non-stress conditions (Yu, 2017; Lin et al., 2020). It is therefore crucial to identify QTL associated with forage yield and quality under water deficit for genetic gain of alfalfa. In this study, we collected 12 traits with the heritability ranging from 0.28 to 0.61 and then performed linkage mapping. However, 48 QTL were detected for only ten traits. There were no QTL mapped for ADF and DM, probably due to the low heritability and no significant differences were observed between the two traits in the two parents. MAS has been one of the most efficient breeding methods due to the advantages of reducing the time and labor required in field tests (Collard and Mackill, 2008; Kumar et al., 2018b). Moreover, quantitative genetics theory predicted that the efficiency of MAS was inversely related to the heritability of the traits (Knapp, 1998; Oladosu et al., 2019). Water scarcity tolerance was a complex trait controlled by polygenes with low to medium heritability (Elena, 2022). Hence, utilizing the QTLs identified in this study, particularly those quality related QTLs of low to moderate heritability, can significantly reduce the time and resources required for breeding efforts.

Transcriptome analysis is an effective method for the identification of differentially expressed stress-responsive genes (Sathik et al., 2018; Liu et al., 2020). Ascertaining the DEGs that change upon water stress in alfalfa is of crucial in understanding the genetic base of drought tolerance. In alfalfa, although drought-responsive DEGs have been reported in previous studies (Ma et al., 2021; Singer et al., 2021), there were some limitations due to the lack of a reference genome. The release of a high-quality alfalfa reference genome has given new power to transcriptional analysis (Chen et al., 2020b). In this study, we aligned the sequencing data to the XingJiangDaYe reference genome and finally obtained a total of 274 DEGs through root and leaf. Through GO enrichment analysis, the most significant enrichment terms were “arabinan catabolic process” and “arabinan metabolic process”, which played a role in maintaining the flexibility of the plant cell wall during water deficit (Moore et al., 2008a; Moore et al., 2008b). In addition, terms related to cell wall biosynthesis such as “xylan catabolic process”, “hemicellulose metabolic process”, and “xylan metabolic process” were also enriched in these DEGs, indicating that changes in the expression of cell wall-related genes were a vital and integral component of alfalfa’s response to water stress. Understanding how the wall adapts to loss of water should provide new insight into crop improvement (Lenk et al., 2019; Ganie and Ahammed, 2021).

Alfalfa is a self-incompatible plant, and the traditional QTL fine mapping and map-based cloning are limited by constructing near-isogenic lines. It has been reported that integration of conventional linkage mapping and RNA-seq can rapidly identify candidates associated with complex traits of interest to replace the fine-mapping process (Park et al., 2019; Derakhshani et al., 2020). In this study, we finally identified 22 drought responsive genes by integrating QTL and DEGs. Plants have evolved complex networks of drought stress response. Abscisic acid (ABA) regulates a number of physiological responses in drought-stressed plants, ensuring a balance of optimal development and stress tolerance (Soma et al., 2021). *MS.gene38710* was annotated as an ABA-responsive element binding factor that can be phosphorylated by upstream genes to regulate the expression of drought-responsive genes (Feng et al., 2019; Zhang et al., 2020). In plants, the phosphatidylethanolamine-binding protein (PEBP) family has been identified to have a crucial role in the regulation of plant growth and developmental processes. Meanwhile, omics data suggested that the PEBP family was also involved in the drought stress response (Manoharan et al., 2016; Li et al., 2017a; Schneider et al., 2019). *OsMFT1*, a member of the PEBP family, has been shown to improve rice drought tolerance by interacting with two key drought-related transcription factors, *OsbZIP66* and *OsMYB26* (Chen et al., 2021). In our study, three PEBP family members (*MS.gene068423*, *MS.gene068413*, and *MS.gene037560*) were identified in two main QTLs, suggesting that they may play an important role in alfalfa drought response. In addition, some differential genes located in the main QTL interval should also be focused on in future research. For example, *MS.gene034021* was annotated as Zein-binding protein, which has not been reported to be related to plant drought resistance. However, it was differentially expressed in G1 and G2 with the high log_2_FoldChange value of -7.40 and -8.36, respectively. Further molecular investigation is needed to illustrate the function of these genes in drought response.

## Conclusion

In the present study, nine main QTLs associated with biomass yield, plant height, and the content of CP, ASH, K, Mg, and P under water deficit condition were identified in the F1 population. The integration of linkage mapping with RNA-seq analysis under water stress, 22 DEGs, excavated from QTL-regions, were identified as potential candidates. The closely linked markers and candidate genes identified in the present study will provide a tool for the MAS breeding program, and new insight for further revealing the molecular mechanism of drought tolerance in alfalfa.

## Data availability statement

RNA-seq raw data used in this study was downloaded from the SRA database in NCBI with the Bioproject accession numbers of PRJNA525327, PRJNA765383, and PRJNA450305.

## Author contributions

QY, JK and ZW conceived and designed the experiments and developed the mapping population. XJ, TY, CW, and Ting Gao collected phenotypic data. XJ, FZ, and AY performed data analysis. XJ and AY wrote the manuscript. L-XY, ZW, and JK revised and finalized the manuscript. All authors contributed to the article and approved the submitted version.

## Funding

This work was supported by the National Natural Science Foundation of China (32071868) and the earmarked fund for China Agriculture Research System (CARS-35-04) and the key research project of Ningxia province for alfalfa breeding program (2019NYY203) and Agricultural Science and Technology Innovation Program (ASTIP-IAS14).

## Conflict of interest

The authors declare that the research was conducted in the absence of any commercial or financial relationships that could be construed as a potential conflict of interest.

## Publisher’s note

All claims expressed in this article are solely those of the authors and do not necessarily represent those of their affiliated organizations, or those of the publisher, the editors and the reviewers. Any product that may be evaluated in this article, or claim that may be made by its manufacturer, is not guaranteed or endorsed by the publisher.

## Supplementary material

Supplementary Figure 1 Frequency distributions of best linear unbiased estimation (BLUE) values of 12 phenotypic data in the F_1_ population.

Supplementary Figure 2 Phenotypic correlations of 12 traits in F_1_ population. Numbers indicate the magnitude of the correlation coefficient. Negatively correlated variables are red, whereas positively correlated variables are blue, with the intensity depending on the magnitude of the correlation. Asterisks indicate the significance level, **P* < 0.05, ***P* < 0.01, ****P* < 0.001.

Supplementary Figure 3 Hierarchical clustering analysis of DEGs in 0h, 6h, and 24h after drought stress. Red, blue and white elements in the matrix indicate up-regulated, down-regulated, and no change genes, respectively.

Supplementary Table 1 Statistical analysis of 12 traits (BLUE values) in two parents and F1 population.

Supplementary Table 2 Consistent QTLs detected in this study. The main QTLs, which were independently identified in a single environment, were considered as consistent QTLs.

Supplementary Table 3 Information of four comparative groups **(G1-G4)**.

Supplementary Table 4 The list of differentially expressed genes (DEGs) in four comparative groups. The DEGs threshold was set at P ≤ 0.01 and |log2 fold change (FC)| > 2.

Supplementary Table 5 GO enrichment analysis of the 4,986 common DEGs in root. The 4,986 DEGs were identified in both G1 and G2.

Supplementary Table 6 GO enrichment analysis of the 1,973 common DEGs in leaf. The 1,973 DEGs were identified in both G3 and G4.

Supplementary Table 7 GO enrichment analysis of the 274 common DEGs among root and leaf. The 274 DEGs were identified in all four comparative groups.

Supplementary Table 8 List of the position information, the log2FoldChange value, and the annotation information of 1,455 genes within nine main QTL regions.
