# Supplementary figures and images for: Identification of QTL and candidate genes associated with biomass yield and Feed Quality in response to water deficit in alfalfa (Medicago sativa L.) using linkage mapping and RNA-Seq
Source: Front Plant Sci. 2022 Oct 17;13:996672. doi: 10.3389/fpls.2022.996672 (PMC9619099; doi:10.3389/fpls.2022.996672)

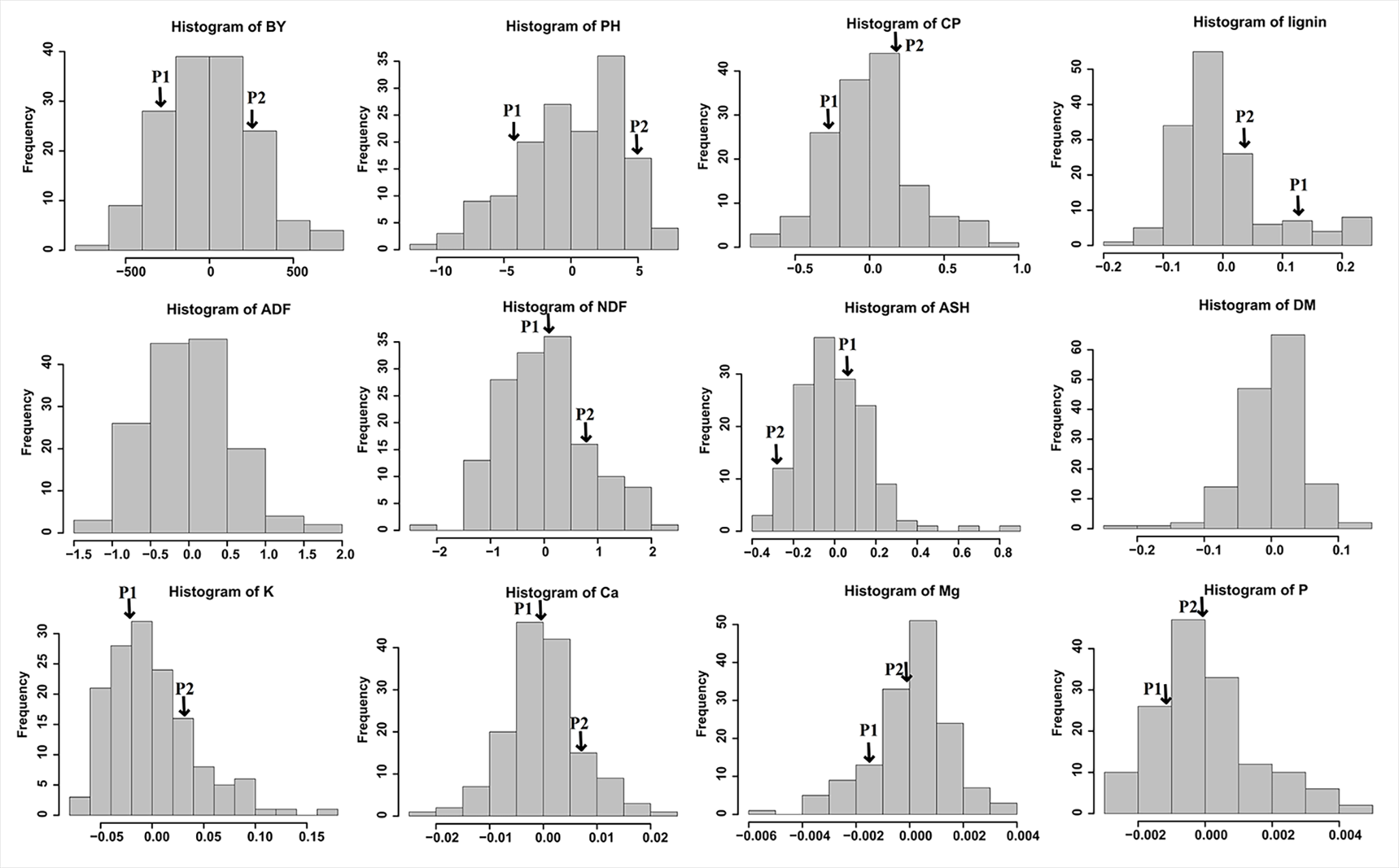

Supplement: Supplementary Figure 1 — Frequency distributions of best linear unbiased estimation (BLUE) values of 12 phenotypic data in the F1 population. [file Image_1.tif]

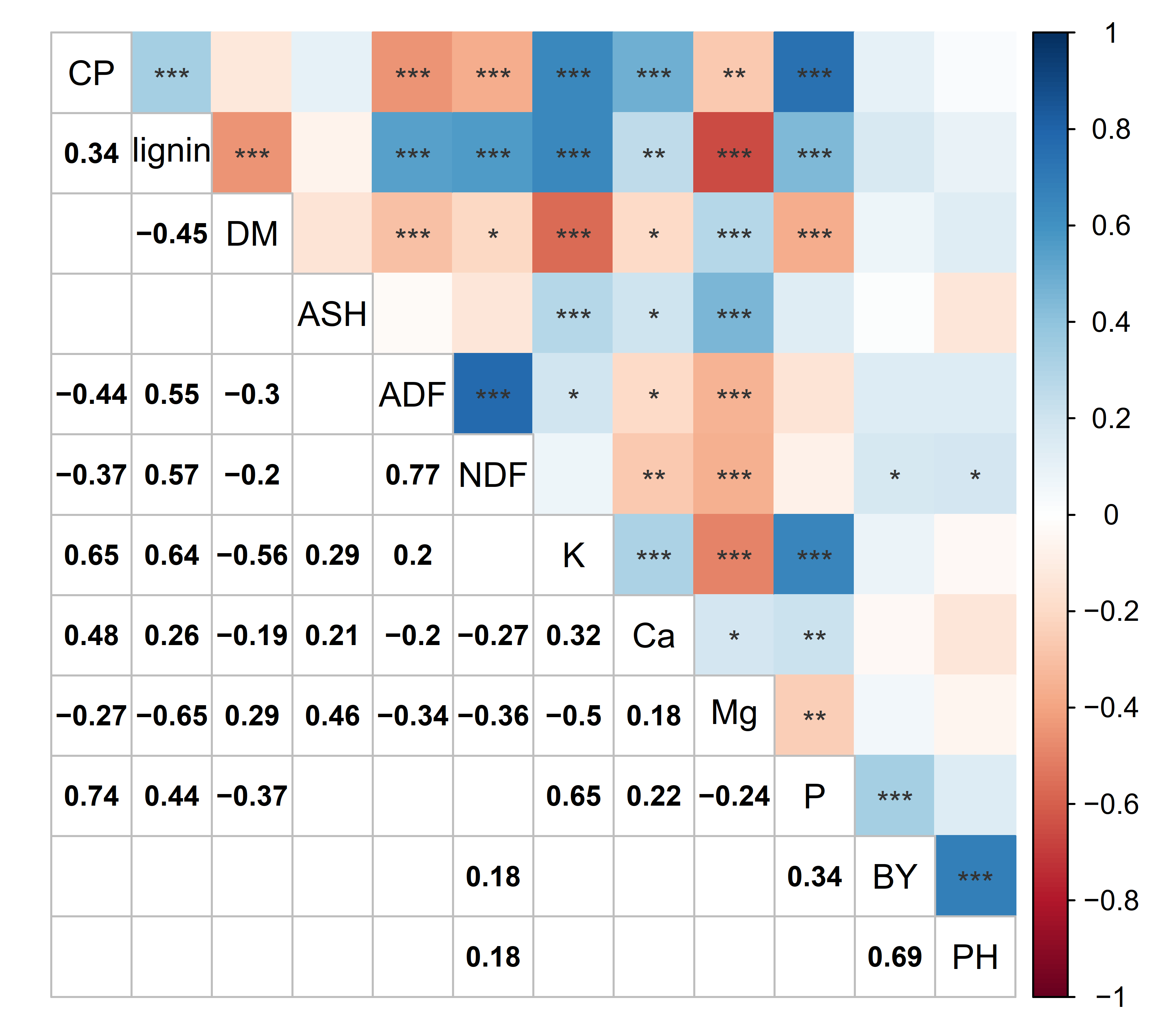

Supplement: Supplementary Figure 2 — Phenotypic correlations of 12 traits in F1 population. Numbers indicate the magnitude of the correlation coefficient. Negatively correlated variables are red, whereas positively correlated variables are blue, with the intensity depending on the magnitude of the correlation. Asterisks indicate the significance level, *P < 0.05, **P < 0.01, ***P < 0.001. [file Image_2.tif]

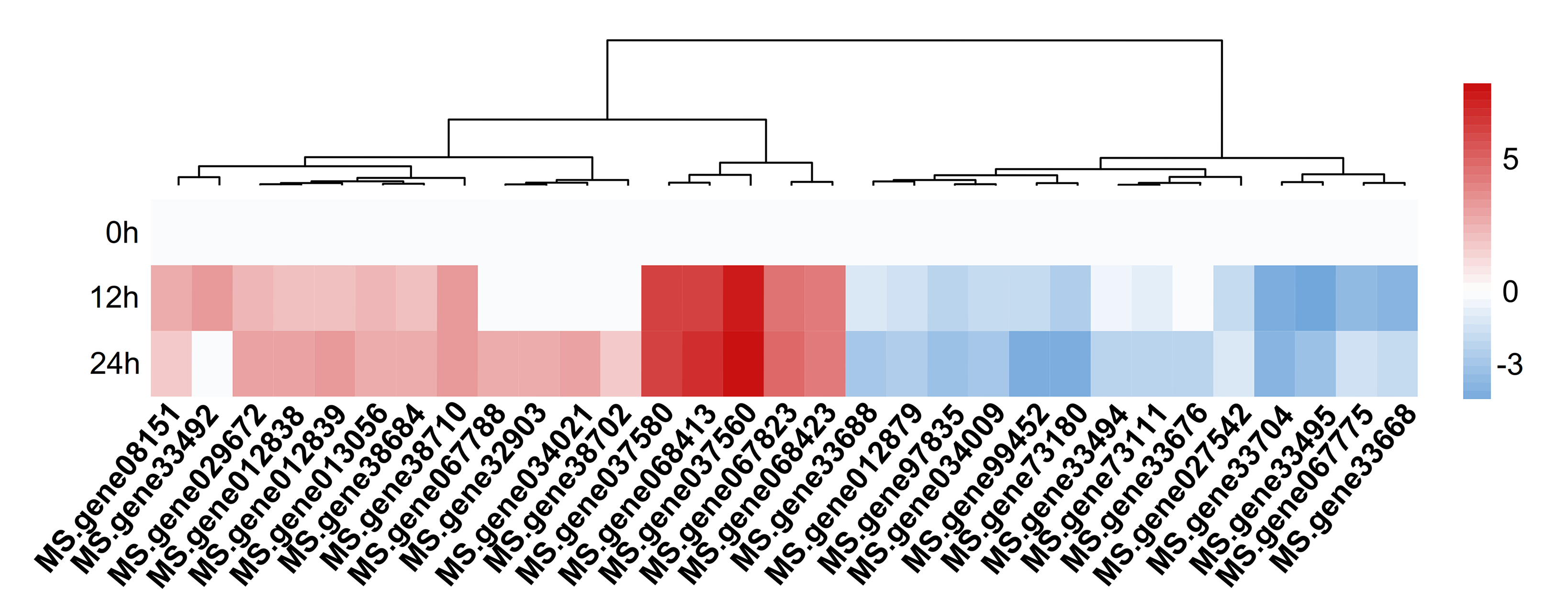

Supplement: Supplementary Figure 3 — Hierarchical clustering analysis of DEGs in 0h, 6h, and 24h after drought stress. Red, blue and white elements in the matrix indicate up-regulated, down-regulated, and no change genes, respectively. [file Image_3.tif]
